# Effects of Age Group, Gender, and Consumption Frequency on Texture Perception and Liking of Cooked Rice or Bread

**DOI:** 10.3390/foods12091793

**Published:** 2023-04-26

**Authors:** Won-Seok Choi, Han-Seok Seo

**Affiliations:** 1 Department of Food Science and Technology, Korea National University of Transportation, Jeungpyeong-gun, Chungbuk 27909, Republic of Korea; 2 Department of Food Science, University of Arkansas, Fayetteville, AR 72704, USA; hanseok@uark.edu

**Keywords:** cooked rice, wheat bread, texture, sensory, demographics

## Abstract

This study aimed to determine whether and how three demographic factors—age group, gender, and consumption frequency—affect texture perception and liking of two staple foods—cooked rice and wheat bread. In total, 346 adults evaluated three cooked rice and four wheat bread samples in terms of three (hardness, stickiness, and chewiness) and four textural attributes (hardness, moistness, chewiness, and softness), respectively, on both 9-point intensity and 5-point Just-About-Right (JAR) scales. Liking of test samples was also rated on 9-point hedonic scales. Age group and gender differed in mean ratings, standard deviations, and JAR responses regarding textural attribute intensity and overall liking of test samples, while the effect of consumption frequency was minimal in this regard. Significant contributors of textural attributes to overall liking of cooked rice and wheat bread differed with age group, gender, and consumption frequency. Effects of age group, gender, and consumption frequency on texture perception and overall liking also varied with test samples. This study provides agricultural and food systems professionals with systematic evidence of how textural attribute perception and liking of foods can change based on demographics and test samples.

## 1. Introduction

Texture is a critical sensory attribute for enjoying food, along with appearance and taste. Excluding beverages, texture has a significant impact (more than 30%) on food preference, particularly in foods such as rice, bread, noodles, meat, potato chips, fruits, and vegetables [1]. Changes in food texture can also affect flavor characteristics through the olfactory pathway [1].

Textural properties are critical in determining food quality, affecting their physicochemical stabilities during processing, storage, and consumption [2,3]. Textural properties also influence consumer perception and liking by formulating texture-related sensory attributes (e.g., hardness, stickiness, crunchiness, and chewiness, etc.) and interacting with other sensory substances (e.g., such as flavor substances) during oral processing [4,5].

Studies have shown individual differences in the perception and liking of textural attributes of foods. Textural perception and liking of foods can vary with ages [6]. Oral physiology and anatomy have been found to change with ages [6,7]. While the number of functional teeth [8] and salivary flow decrease [9,10], the number of tooth wears or tooth implants [11] and the muscle activity required for biting and swallowing increases with age [12,13]. The ability to recognize shapes and orientation of the food bolus [14] and coordinate the oral devices [15] in the mouth declines. Such aging-induced changes in oral processing behavior can increase the number of chews and time required for swallowing [7,13] and the size of the swallowed food bolus [16]. These changes may result in overall differences between younger and older people in perception, liking, and behavior toward foods [7].

Gender is another influencing factor. Kälviäinen et al. (2000) demonstrated that middle-aged males, compared to middle-aged females, preferred harder and more adhesive candy textures; additionally, they preferred starch-based candies, while the middle-aged females preferred pectin-based samples [17]. These empirical variations might be associated with differences in oral processing behavior. Males have been reported to have a higher maximal biting force (approximately 30% higher in a study by [18,19]) and salivary flow [10] and larger bite sizes [19], resulting in a shorter time required for swallowing and higher eating rates [7,19]. Males were more sensitive to hardness intensity than females in jelly samples [20].

The consumption frequency of a specific food also affects texture perception and liking [17,21,22]. Although people are typically likelier to prefer food samples that they are more familiar with, this trend has been inconsistent [17], suggesting that further research is needed on the effect of consumption frequency on texture perception and liking of foods.

Rice (*Oryza sativa* L.) has been cultivated in Asia for a long time and is one of the world’s top major crops, along with wheat and corn. It is a typical staple food crop that supplies over a quarter of the required calories to about half of the world’s population [23]. Texture is the most critical sensory factor affecting rice preference [24].

Cooked rice and wheat bread were selected as test samples because they are popular dietary staples in many countries, their texture has a significant impact on food preference, and their major textural attributes are different. Both items are popular in the Republic of Korea, although cooked rice is considered a staple for most of the population compared to wheat bread. However, as the Korean diet has become westernized, eating wheat bread and its various forms (e.g., toasts and sandwiches) has become more common in Korea [25].

Previous studies have investigated the effects of age group, gender, and consumption frequency on not only texture perception but also perceptions of other sensory attributes related to appearance, flavor, and taste [4,26,27]. Given that questions related to perceptions of other sensory cues may influence participants’ attention and perception towards the textural aspects of foods, this study was designed to exclusively examine participants’ perception and liking of the texture of the target foods by excluding questions associated with other sensory attributes in a main test. Furthermore, this study, which included a sizable sample of 346 participants, systematically investigated the effects of three factors on texture perception and liking of two food items compared to previous studies [6].

Thus, this study aimed to investigate how age group, gender, and consumption frequency affect texture perception and overall liking of two foods staples: cooked rice and wheat bread. The research aims to contribute to a better understanding of how consumers’ sensory preferences towards these foods can be influenced by these factors.

## 2. Materials and Methods

The protocol used in this study was approved by the Institutional Review Board of Korean National University of Transportation (KNUT IRB 2019-18 and 2020-20, Jeungpyeong-gun, Chungbuk, Republic of Korea). Prior to participation, the experimental procedure was explained, and informed written consent was obtained from each participant.

### 2.1. Participants

In total, 346 adults (172 females and 174 males) aged between their 20s and 70s, who were self-reported habitual eaters of cooked rice and wheat bread (eat each item at least once a week), were recruited through a consumer profile database of Sensometrics Co., Ltd. (Seoul, Republic of Korea) over 2 years. The experiment was conducted on healthy individuals aged 20–70 years with no abnormalities in chewing or swallowing function who were willing to participate. Prior to the sensory evaluation, participants were advised to refrain from smoking, drinking alcohol, and taking medication that could affect their taste and texture evaluations. Table 1 shows the participants’ demographics. There were four age groups: 20–29, 30–49, 50–69, and 70–79 years old. The subcategories of consumption frequency were combined and classified into three groups: “low”, “medium”, and “high”.

### 2.2. Test Samples and Preparation

Microwavable cooked rice products (*n* (brand) = 3) and wheat bread products (*n* (brand) = 4) were used as test samples and purchased from local supermarkets (Seoul, Republic of Korea). From the cooked rice and wheat bread products sold in Korea, three brands of cooked rice and four brands of wheat bread were selected as test samples. Microwavable cooked rice products were selected to avoid any variations in sample preparation. Each cooked rice sample (210 g) was heated for 2 to 2.5 min in a microwave oven (MW22CD9, LG Electronics Tianjin Appliances Co., Ltd., Tianjin, China) according to the manufacturer’s cooking directions. Twenty-five grams of the cooked rice was placed in a 70 mL polystyrene cup (Daeheungpojang, Corporation, Kimpo, Gyeonggi-do, Republic of Korea) identified with a random three-digit code. For wheat bread, after removing the crust of each sample, it was sliced into halves, and a half slice (∼96 mm × 56 mm × 14 mm) was served on a plastic plate.

### 2.3. Procedure

This study was conducted in individual sensory booths at the Sensory Evaluation Center at Sensometrics Co., Ltd. (Seoul, Republic of Korea). Prior to sample presentation, an orientation session detailing both the experimental protocol and scaling was conducted for each participant. Instructions and scales were presented using the sensory analysis software, SensMine (Sensometrics Co., Ltd.).

#### 2.3.1. Cooked Rice Samples

Each participant was presented with three cooked rice samples, along with a white plastic spoon, at 70 °C (standard deviation = 2 °C) in a sequential monadic fashion consistent with the Williams Latin Square design [28]. To ensure that the participants’ rating patterns did not influence the effects of age group, gender, or consumption frequency on ratings of texture perception and liking [29], participants were asked to rate the visual glossiness of the cooked rice samples on a 9-point category scale ranging from 1 (extremely low) to 9 (extremely high) and a 5-point Just-About-Right (JAR) scale (1 = much too light/little, 3 = JAR, and 5 = much too dark/much). Similarly, participants were asked to evaluate the three cooked rice samples in terms of three textural attributes—hardness, stickiness, and chewiness—on both the scales. Finally, the overall liking of each cooked rice sample was rated on a 9-point hedonic scale ranging from 1 (dislike extremely) to 9 (like extremely). A 90 s break was allowed between sample presentations. During the break, alkaline water (ICIS 8.0, Lotte Chilsung Beverage Co. Ltd., Seoul, Republic of Korea) was presented for palate cleansing.

#### 2.3.2. Wheat Bread Samples

Following a break for 30 min after the sensory evaluation of cooked rice samples, each participant was presented with four wheat bread samples in a sequential monadic fashion consistent with the Williams Latin Square design. Participants were asked to rate wheat bread samples in terms of visual color and textural attributes—hardness, moistness, chewiness, and softness—on both a 9-point category scale and a 5-point JAR scale. The overall liking of each wheat bread sample was rated on a 9-point hedonic scale. A 90 s break was given between sample presentations. During the break, alkaline water was presented for palate cleansing.

### 2.4. Data Analysis

Data analysis was performed using SPSS 28.0 for Window^TM^ (IBM SPSS Inc., Chicago, IL, USA) and XLSTAT software (Addinsoft, New York, NY, USA). To determine whether ratings of attribute intensity or overall liking for cooked rice or wheat bread samples differed as a function of the three factors—age group, gender, and consumption frequency—a three-way analysis of variance (ANOVA) was performed. If a significant difference was found among the test samples, post hoc multiple pairwise comparisons were performed using Tukey’s Honestly Significant Difference, Scheffe, or Games–Howell tests, depending on the condition. When the subgroup sizes of each factor were unequal, the harmonic mean method [30] was used. In the same manner, a three-way ANOVA, treating “age group”, “gender”, and “consumption frequency” as fixed effects, was performed to test whether standard deviations of the intensity or liking ratings differed for cooked rice or wheat bread samples. Stepwise multivariate regression analysis was also performed to determine the contribution of intensity ratings of each textural attribute on the overall liking of the test samples as a function of age group, gender, and consumption frequency.

For JAR scale data, chi-squared tests were conducted to test whether proportions of response categories for cooked rice or wheat bread samples differed as a function of age group, gender, or consumption frequency. A penalty analysis was also performed to identify how much each textural attribute affected the overall liking of cooked rice or wheat bread samples. A statistically significant difference was defined as *p* < 0.05.

## 3. Results

### 3.1. Effects of Age Group, Gender, and Consumption Frequency on Texture Perception and Liking of Cooked Rice Samples

No significant differences were found among the three cooked rice samples in the non-textural attribute (i.e., visual glossiness) as a function of age group (*p* = 0.15), gender (*p* = 0.17), or consumption frequency (*p* = 0.86). Thus, the participants’ scale-usage style had minimal impact on this study’s results [29,31].

**Figure 1:**
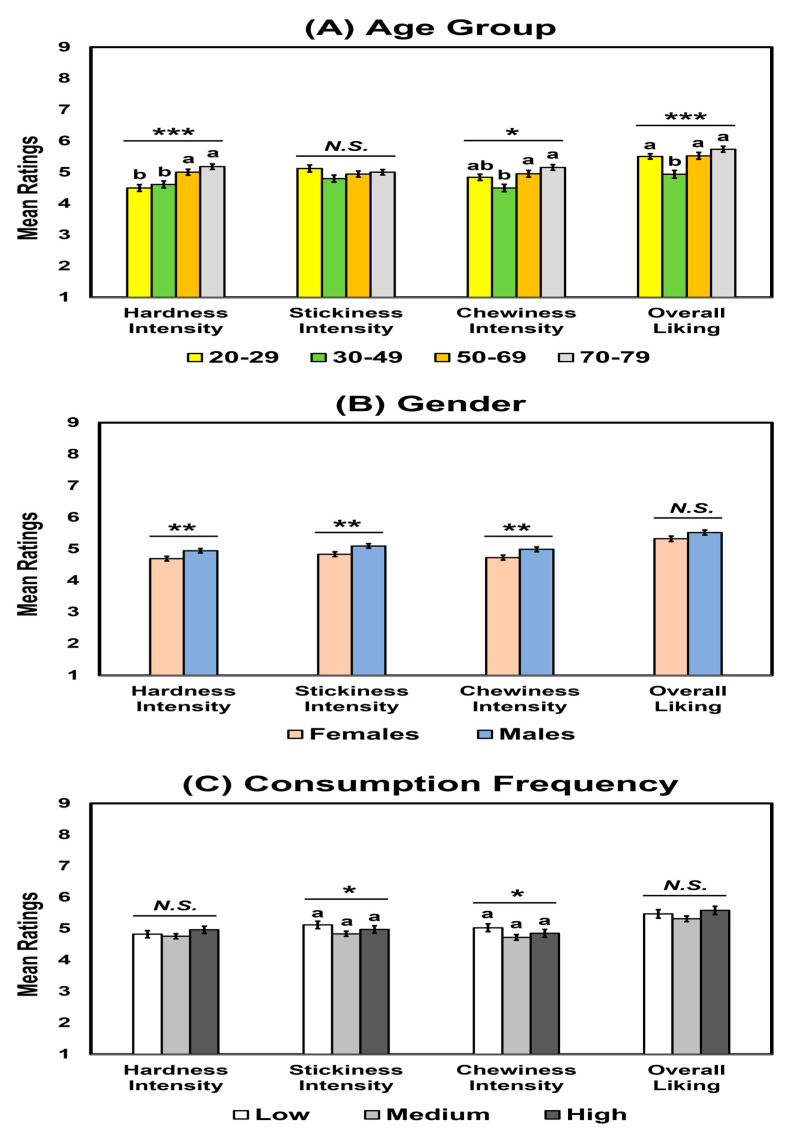
Mean comparisons of hardness, stickiness, and chewiness intensities and overall liking of cooked rice samples as a function of age group (**A**), gender (**B**), or consumption frequency of cooked rice (**C**). Error bars represent standard errors of the means. Means with different letters within each attribute intensity or liking indicate a significant difference at *p* < 0.05. *N.S.* indicates no significance at *p* < 0.05. *, **, and *** represent a significant difference at *p* < 0.05, *p* < 0.01, and *p* < 0.001, respectively.

As shown in Table 2, no significant interactions were found among the three factors regarding hardness intensity, stickiness intensity, chewiness intensity, or overall liking (*p* > 0.05), except for an interaction between age group and consumption frequency in hardness intensity. Table 2 and Figure 1A show that age group significantly affected hardness intensity (*p* < 0.001), chewiness intensity (*p* = 0.03), and overall liking (*p* < 0.001) but not stickiness intensity (*p* = 0.16). The 50–69 and 70–79 age groups exhibited higher ratings of hardness intensity than the 20–29 and 30–49 age groups. The 50–69 and 70–79 age groups also gave higher ratings of chewiness intensity than the 30–49 age groups. The 30–49 age group exhibited the lowest ratings of overall liking for cooked rice samples. Table 2 and Figure 1B display that males exhibited higher intensity ratings for hardness (*p* = 0.004), stickiness (*p* = 0.004), and chewiness (*p* = 0.002) than females, while no gender difference was observed for overall liking of cooked rice samples (*p* = 0.09). Finally, as shown in Table 2 and Figure 1C, while consumption frequency of cooked rice affected stickiness intensity (*p* = 0.02) and chewiness intensity (*p* = 0.04), post hoc tests revealed no significant differences among the subgroups. There were no significant differences in hardness intensity (*p* = 0.37) and overall liking (*p* = 0.15) as a function of consumption frequency.

**Figure 2:**
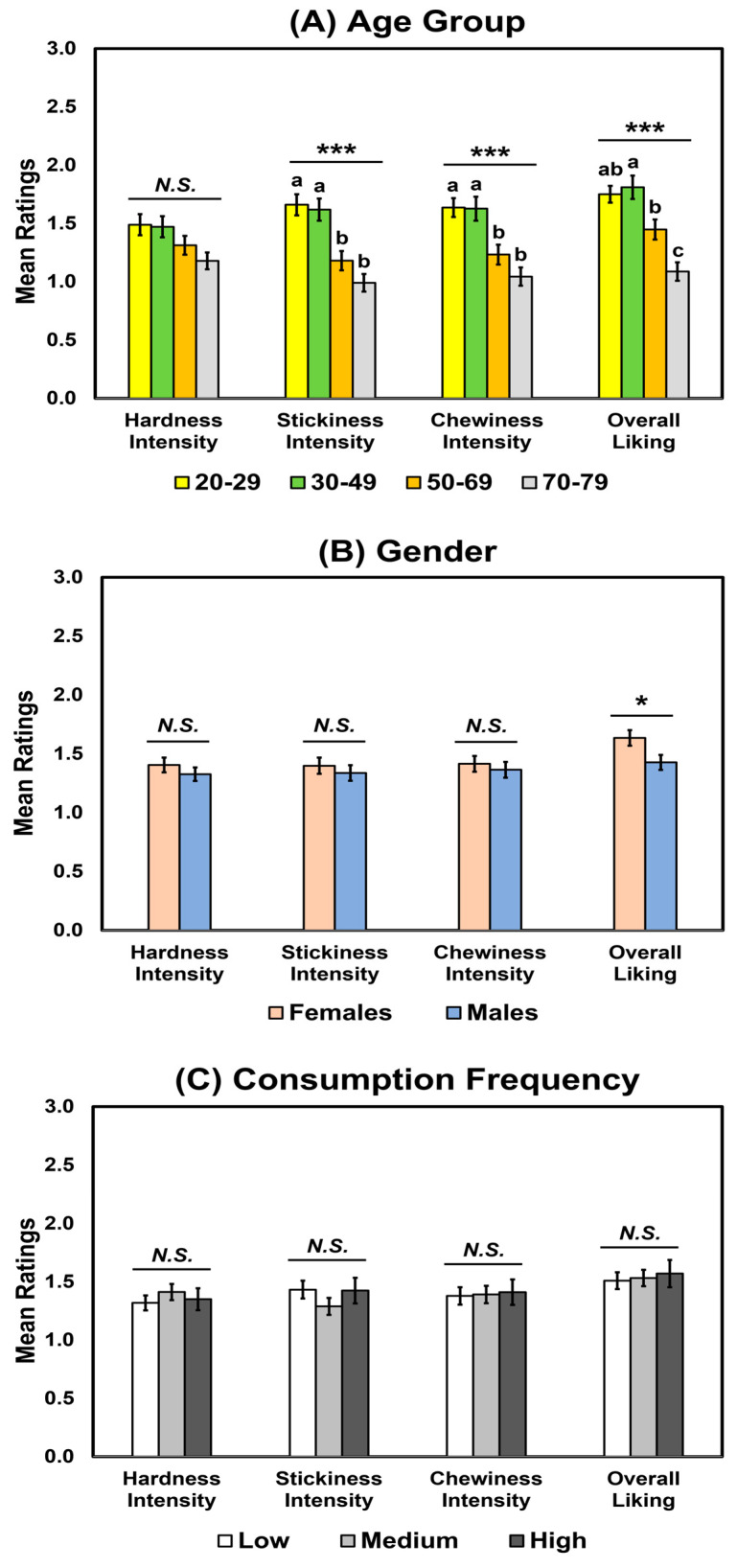
Mean comparisons of the standard deviations of the ratings for hardness, stickiness, and chewiness intensities and overall liking of cooked rice samples as a function of age group (**A**), gender (**B**), or consumption frequency of cooked rice (**C**). Error bars represent standard errors of the means. Means with different letters within each attribute intensity or liking indicate a significant difference at *p* < 0.05. *N.S.* indicates no significance at *p* < 0.05. * and *** represent a significant difference at *p* < 0.05 and *p* < 0.001, respectively.

For standard deviations of the intensity or liking ratings among the three cooked rice samples, no significant interactions were found among the three factors: age group, gender, and consumption frequency (*p* > 0.05; Table 3). Age group affected stickiness (*p* < 0.001) and chewiness intensities (*p* < 0.001) and overall liking (*p* < 0.001) but not hardness intensity (*p* = 0.12). The 20–29 and 30–49 age groups exhibited larger standard deviations among the three cooked rice samples than the 50–69 and 70–79 age groups regarding stickiness and chewiness intensities (Figure 2A). For overall liking, compared to other age groups, the oldest age group (70–79 years) exhibited smaller standard deviations among the three cooked rice samples (Figure 2A). Females showed larger standard deviations among the three cooked rice samples than males regarding overall liking (*p* = 0.01; Table 3), while no significant gender differences were observed in hardness (*p* = 0.18), stickiness (*p* = 0.61), and chewiness (*p* = 0.66) intensities. Finally, there were no significant effects of consumption frequency on standard deviations among the three cooked rice samples in terms of hardness (*p* = 0.65), stickiness (*p* = 0.21), and chewiness (*p* = 0.95) intensities and overall liking (*p* = 0.97) (Table 3).

Table 4 represents a summary of the multivariate stepwise linear regression for predicting overall liking of cooked rice samples using the three textural attributes—hardness, stickiness, and chewiness—as a function of age group, gender, and consumption frequency. For age group, chewiness intensity was a key modulator in overall liking of cooked rice across the four age groups. For the 20–29 age group, stickiness intensity was not a contributor to determining overall liking of cooked rice samples. For the 70–79 age group, hardness intensity and stickiness intensity were found to be non-significant contributors to overall liking of cooked rice samples. Regarding gender, while all three attributes were significant contributors to overall liking of cooked rice samples among females, hardness and chewiness intensities, not stickiness intensity, were identified as significant contributors in the model among males. With respect to consumption frequency, all three attributes were identified as significant contributors to overall liking of cooked rice samples among participants with a low frequency of cooked rice consumption. While hardness and chewiness intensities were significant contributors to overall liking of cooked rice samples among participants with a medium frequency of cooked rice consumption, stickiness and chewiness intensities were determined as significant contributors among those with a high frequency of cooked rice consumption.

Table 5 shows the percentages and mean drops of the “too little” or “too much” responses on the 5-point JAR scale for hardness, stickiness, and chewiness of cooked rice samples as a function of age group, gender, and consumption frequency. When reviewing 20% or above 20% of the total participants in Table 5, the hardness intensities of the test samples were perceived lower than what the participants expected across age groups, gender, and consumption frequency, except for “too much” hardness among the 70–79 age group (20.33%). Similarly, the stickiness and chewiness intensities of the test samples were perceived lower than what they expected across age groups, gender, and consumption frequency, except for “too much” stickiness among the 20–29 age group (24.52%) and among males (20.88%).

### 3.2. Effects of Age Group, Gender, and Consumption Frequency on Texture Perception and Liking of Wheat Bread Samples

Significant differences were found among the four wheat bread samples in the non-textural attribute (i.e., color intensity) as a function.

**Figure 3:**
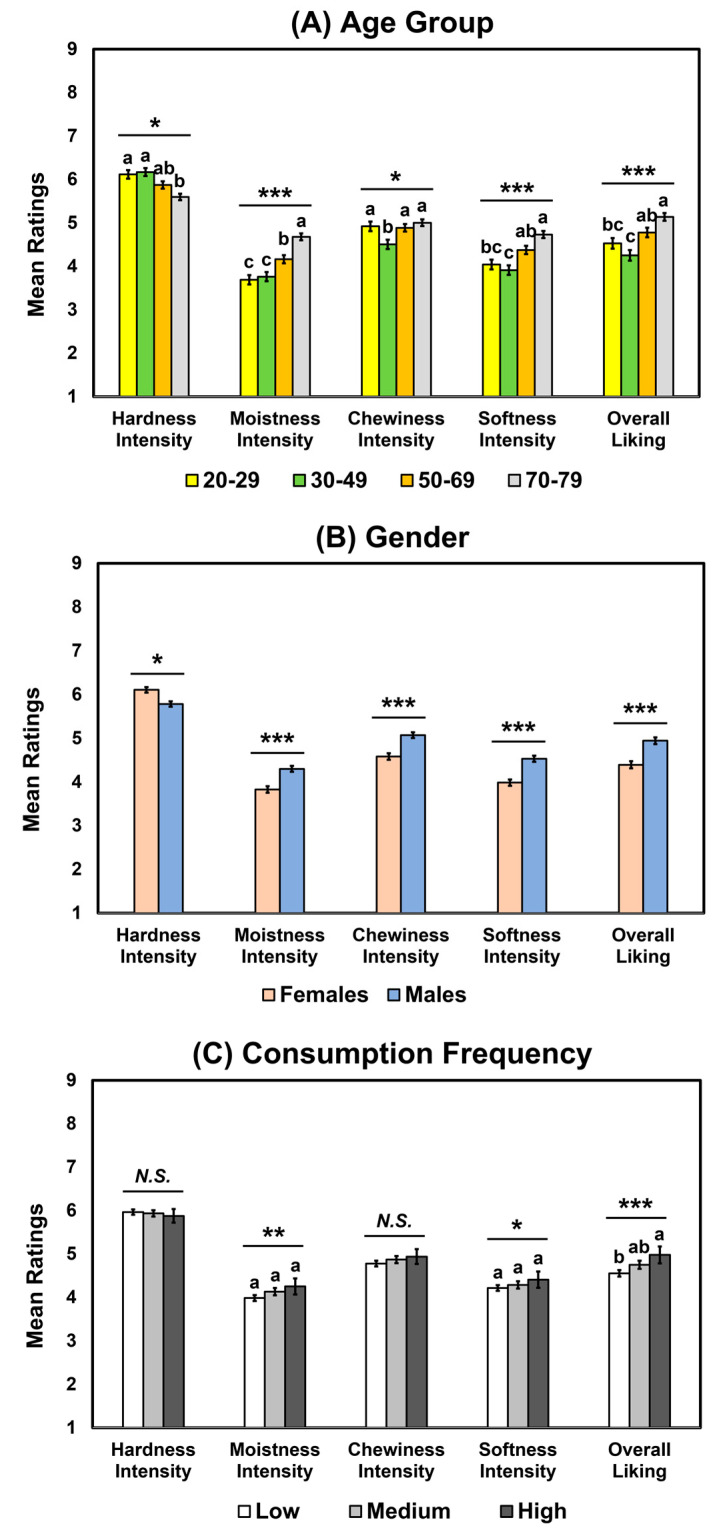
Mean comparisons of hardness, stickiness, and chewiness intensities and overall liking of wheat bread samples as a function of age group (**A**), gender (**B**), or consumption frequency of wheat bread (**C**). Error bars represent standard errors of the means. Means with different letters within each attribute intensity or liking indicate a significant difference at *p* < 0.05. *N.S.* indicates no significance at *p* < 0.05. *, **, and *** represent a significant difference at *p* < 0.05, *p* < 0.01, and *p* < 0.001, respectively.

As shown in Table 6, significant interactions were observed between age group and gender regarding the hardness (*p* = 0.005) and moistness intensities (*p* = 0.02). No other significant interactions were observed among the three factors (*p* > 0.05). Table 6 and Figure 3A demonstrate that age group affected the hardness (*p* = 0.02), moistness (*p* < 0.001), chewiness (*p* = 0.03), and softness (*p* < 0.001) intensities and overall liking (*p* < 0.001). For hardness intensity, the 20–29 and 30–39 age groups exhibited higher ratings than the 70–79 age group. In contrast, the 70–79 age group exhibited the highest ratings of moistness intensity, while the 20–29 and 30–39 age groups showed the lowest ratings in this regard. The 70–79 age group had higher ratings of softness intensity than the 20–29 and 30–49 age groups. For chewiness intensity, the 30–49 age group exhibited lower ratings than the other age groups. The 70–79 age group exhibited higher ratings of overall liking than the 20–29 and 30–49 age groups, and the 50–69 age group showed higher ratings of overall liking than the 30–49 age group. Regarding gender, males exhibited lower ratings of hardness intensity than females (*p* = 0.045), but higher ratings of moistness (*p* < 0.001), chewiness (*p* < 0.001), and softness intensities (*p* < 0.001) and overall liking (*p* < 0.001) (Table 6 and Figure 3B). Finally, while consumption frequency affected moistness (*p* = 0.002) and softness intensities (*p* = 0.03), post hoc tests found no significant differences at *p* < 0.05, as shown in Figure 3C. No significant effects of consumption frequency were found on the hardness (*p* = 0.24) and chewiness intensities (*p* = 0.16) of wheat bread samples. However, consumption frequency affected the overall liking of wheat bread samples (*p* < 0.001). Participants with a high frequency of bread consumption exhibited higher likings of the wheat bread samples than those with a low frequency of bread consumption (Figure 3C).

**Figure 4:**
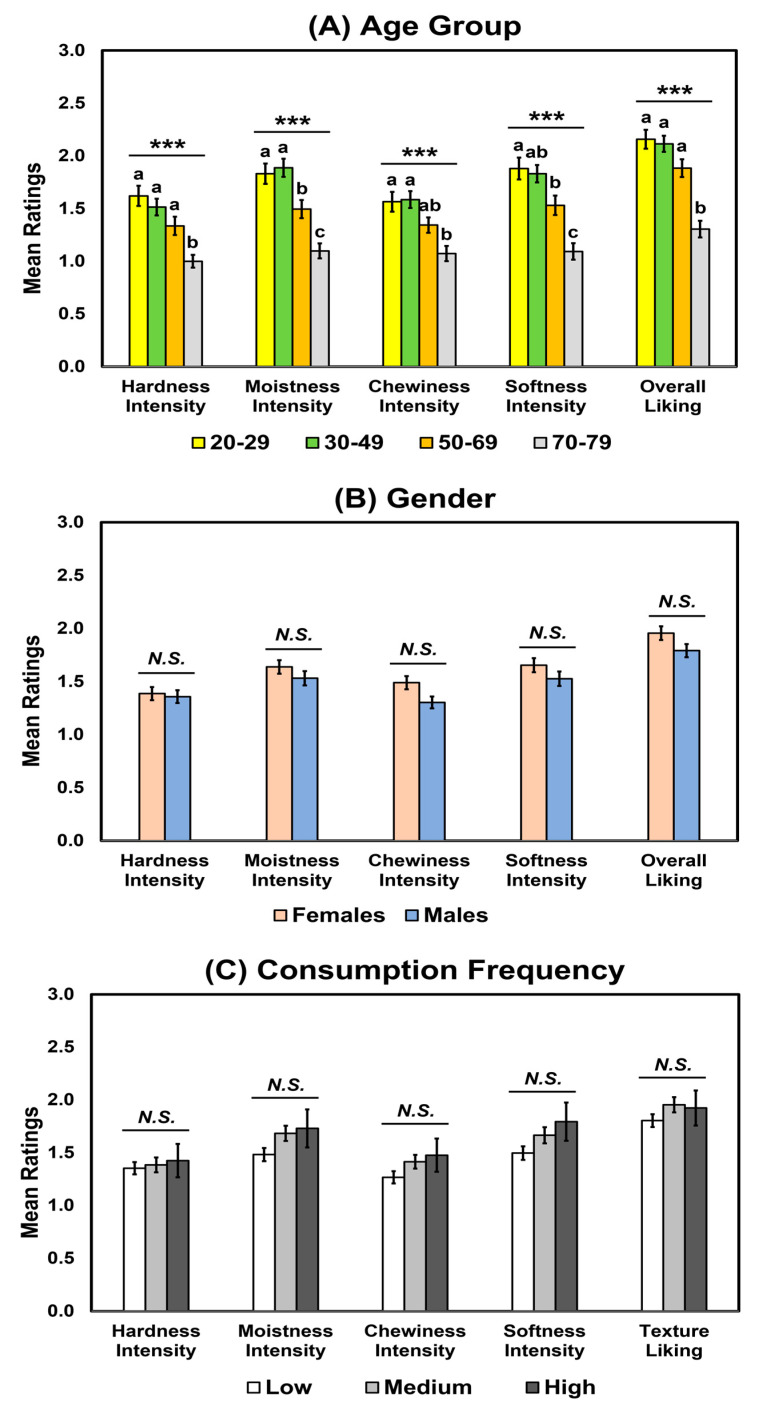
Mean comparisons of the standard deviations of the ratings for hardness, moistness, chewiness, and softness intensities and overall liking of wheat bread samples as a function of age group (**A**), gender (**B**), or consumption frequency of wheat bread (**C**). Error bars represent standard errors of the means. Means with different letters within each attribute intensity or liking indicate a significant difference at *p* < 0.05. *N.S.* indicates no significance at *p* < 0.05. *** represent a significant difference at *p* < 0.001.

For standard deviations of the intensity and liking ratings among the four wheat bread samples, there were no significant interactions of gender with age group or consumption frequency (*p* > 0.05), while there was a significant interaction between age group and consumption frequency in the moistness (*p* = 0.002), chewiness (*p* = 0.02), and softness intensities (*p* = 0.004) and overall liking (*p* = 0.001) (Table 7). Age group affected the hardness (*p* < 0.001), moistness (*p* < 0.001), chewiness (*p* < 0.001), and softness intensities (*p* < 0.001) and overall liking (*p* < 0.001). As shown in Figure 4A, the 70–79 age group exhibited the smallest standard deviations in these ratings, while the younger age groups (e.g., 20–29 and 30–49) were likely to have larger standard deviations. Younger participants have greater variability in their texture preferences than older participants, who have more consistent preferences for certain textural attributes [32,33,34]. However, neither gender nor consumption frequency affected texture perception or overall liking of the wheat bread samples (Table 7 and Figure 4).

Table 8 summarizes the multivariate stepwise linear regression for predicting overall liking of wheat bread samples using the four textural attributes—hardness, moistness, chewiness, and softness—as a function of age group, gender, and consumption frequency. Regarding age group, while moistness intensity was the most important contributor to increasing overall liking of wheat bread samples in three age groups (20–29, 30–49, and 50–69), softness intensity was the most critical contributor in the 70–79 age group. For gender, the softness and moistness intensities were the most important contributors to increasing overall liking of wheat bread samples for females and males, respectively. Regarding consumption frequency, softness intensity was the most important contributor to the overall liking of wheat bread samples among participants with a low or medium frequency of wheat bread consumption, but it was not a significant contributor among those with a high consumption frequency. For those with a high frequency of wheat bread consumption, moistness intensity was the most important contributor in this regard.

Table 9 shows percentages and mean drops of the “too little” or “too much” responses on the 5-point JAR scale for hardness, moistness, chewiness, and softness of wheat bread samples as a function of age group, gender, and consumption frequency. When reviewing 20% or above 20% of total participants in Table 9, the hardness intensities of the test samples were perceived higher than what the participants expected across age groups, gender, and consumption frequencies. In contrast, the moistness, chewiness, and softness intensities of the wheat bread samples were perceived lower than what they expected across age groups, gender, and consumption frequency. While participants with a “high” consumption frequency rated the wheat bread samples as either “too little” (37.50%) or “too much” (22.66%), the mean drops for “too little” (1.91) were greater than those for “too much” (0.67).

## 4. Discussion

While previous studies have shown the effects of demographic profiles on texture perception or liking of test food samples [4,26,27], most studies did not focus solely on the textural aspects of foods, and their sample sizes were not large enough to draw a conclusion. With such knowledge gaps, this study, which had a large sample size (*n* = 346), was performed to determine how three key demographic factors, age group, gender, and frequency of consumption of target foods, affect the intensity of textural attributes and overall liking of two commonly consumed food items whose texture is critical to quality: cooked rice and wheat bread.

### 4.1. Effects of Age Group on Texture Perception and Liking of Cooked Rice or Wheat Bread Samples

Age group played an important role in modulating the textural attribute intensities and overall liking of cooked rice or wheat bread samples, supporting previous studies. The hardness or chewiness intensities and overall liking significantly differed among the four age groups. When compared to the younger age groups (i.e., 20–29 and 30–49), the 70–79 age group exhibited higher ratings of hardness intensity for cooked rice samples, while the group’s ratings of the hardness intensity of wheat bread samples were lower (Table 2 and Table 6 and Figure 1 and Figure 3). This result was consistent with the results of the JAR ratings (Table 5 and Table 9), where more participants in the younger age groups considered the cooked rice and wheat bread samples as “too little” and “too much”, respectively, than those in the 70–79 age group. These findings suggest that the age group-induced difference in hardness intensity is relative, not absolute. Thus, the effect of age group on hardness intensity may be related not just to differences in oral physiology and anatomy, but also to an individual’s previous experience with the test samples [4,6,7,8,9,11,12,13,16,35]. In particular, the 70–79 age group differs from the other age groups because their previous experiences also had a significant impact on food texture perception and liking, as has been found in other studies [36,37].

In contrast, the 30–49 age group exhibited lower intensities of chewiness than other age groups in both cooked rice and wheat bread samples (Figure 1 and Figure 3). Similarly, the percentages of participants for “too little” on the JAR scales for cooked rice and wheat bread samples were higher in the 30–49 age group than the other groups (Table 5 and Table 9), reflecting that individuals’ expectations of chewiness or physical/physiological characteristics required for chewing could be different in the 30–49 age group over the lifespan.

The standard deviations of the intensity or liking ratings were smaller at younger ages (Figure 2 and Figure 4), similar to previous findings. These findings suggest that individual variations in texture perception and liking diminish as individuals become older, which might be related to an aging-reduced sensitivity of sensory functions. Additionally, individuals’ responses to textural attributes across different categories of food decreased as age increased [37].

The textural attributes influencing the overall liking of cooked rice or wheat bread samples differed with age groups. For cooked rice, while chewiness intensity was identified as a key driver of liking across all age groups, hardness or stickiness intensity was not considered as a significant contributor to increasing the overall liking of cooked rice for the youngest (20–29) and oldest (70–79) age groups (Table 4), contradicting previous studies reporting that overall liking of cooked rice exhibited strong positive and negative correlations with stickiness and hardness, respectively, among Korean participants [38]. This may be due to the analysis based on the first-order regression equation with a low R^2^ value. For wheat bread, while moistness intensity was identified as the most important contributor in the 20–29, 30–49, and 50–69 age groups, its impact was smaller in the 70–79 age group, with softness intensity as the most important driver of liking for wheat bread samples (Table 8).

### 4.2. Effects of Gender on Texture Perception and Liking of Cooked Rice or Wheat Bread Samples

Gender partially affected the texture perception and liking of cooked rice or wheat bread samples (Figure 1 and Figure 3). Compared to females, males exhibited higher intensity ratings of textural attributes in both cooked rice and wheat bread samples, except for hardness intensity for wheat bread samples. The overall liking of wheat bread samples was higher in males than in females, but such gender difference in overall liking was not observed in the cooked rice samples.

Overall, gender did not affect individual variations in the intensity ratings of texture attributes (Figure 2 and Figure 4) or influence JAR ratings for cooked rice or wheat bread samples (Table 5 and Table 9). Although previous research has reported that females were likely to be more texture-oriented, while males seem to be more flavor-oriented [37,39], such a gender difference was not pronounced in this study. While Luckett and Seo [37] demonstrated that females used “crunch/crunchy” and “crisp/crispy” words more frequently than males when eliciting word responses to the name and image of different food items, it should be noted that cooked rice or wheat bread samples tested in the present study were not related to such texture attributes [37]. Thus, further research is needed to determine the effect of gender on texture perception and liking of crunchy or crispy foods.

As shown in Table 4 and Table 8, there were gender differences in determining the significant contributors of textural attributes to increasing the overall liking of cooked rice or wheat bread samples. While chewiness was the most important attribute for the overall liking of cooked rice in both females and males, the impact of stickiness intensity on overall liking was observed only for females. Softness intensity was the most important driver of liking for wheat bread samples among females, while moistness intensity was the key driver among males.

### 4.3. Effects of Consumption Frequency on Texture Perception and Liking of Cooked Rice or Wheat Bread Samples

The consumption frequency of cooked rice or wheat bread affected the intensity of textural attributes and the overall liking of the samples. However, for cooked rice, there was no significant difference in stickiness and chewiness intensity, and for wheat bread, there was no significant difference in moistness and softness intensity based on frequency, as indicated by the post hoc tests (Figure 1C and Figure 3C). There were no variation differences in the intensity of textural attributes or the overall liking of each sample due to differences in consumption frequency (Figure 2 and Figure 4), and the percentages of “too little” or “too much” responses of the JAR scale for textural attributes (Table 5 and Table 9) were also minimal. Little effect of consumption frequency on texture perception or liking has been observed in past studies using other food items [17], although contrasting results (i.e., a significant effect) have also been reported [21,22].

The contributors of textural attributes to the overall liking of cooked rice or wheat bread samples were different as a function of consumption frequency. For the overall liking of cooked rice, while chewiness intensity was identified as the most important contributor across the three subgroups, stickiness intensity and hardness intensity did not contribute to the overall liking for participants with the medium and high frequencies of rice consumption, respectively (Table 4). For the overall liking of wheat bread, while softness intensity was the most important contributor in both low and medium subgroups of consumption frequency, it was identified as a non-significant contributor in the high subgroup with moistness intensity as the most important contributor (Table 8).

### 4.4. Implications and Limitations

Since this study clearly shows how age group, gender, and consumption frequency differed regarding the texture perception or liking of the cooked rice and wheat bread samples, professionals in agricultural and food systems can employ the findings to optimizations in breeding and post-harvest processing of rice or wheat and to product development [26,40,41,42], increasing consumer acceptance and sales of the target products. Additionally, product developers and sensory professionals can gain a better understanding of how to develop cooked rice or wheat bread products for their target consumers, such as the elder adults, females or males, and frequent or infrequent eaters [37,39].

This study has certain limitations. Although multiple samples were tested for either cooked rice or wheat bread, the samples are classified into similar types of cooked rice (e.g., short grain) or wheat bread (e.g., thin-sliced wheat bread). Even though these types of cooked rice or wheat bread are often consumed in Korea and Japan, it would be interesting to conduct further research with cooked rice or wheat bread samples having a wider spectrum of textural properties [27,43].

This study uses a relatively larger sample size (*n* = 346) across age group, gender, and consumption frequency than other studies, when considering earlier research in which cultural or regional background influenced texture perception or liking of cooked rice or bread [26,27,40], it would be worth identifying how such variables modulate the textural attribute intensities and overall liking of test samples. In a cross-cultural study by Choi et al. [27], chewiness was a negative driver of liking for cooked rice samples among U.S. participants, while stickiness was a positive driver of liking among Korean participants [27].

## 5. Conclusions

The age group played an important role in modulating the intensities of textural attributes and overall liking of cooked rice or wheat bread samples, supporting previous studies. These findings suggest that the age group-induced difference in hardness intensity is relative, not absolute. Thus, the effect of age group on hardness intensity may be related not just to differences in oral physiology and anatomy, but also to an individual’s previous experience with the test samples. The standard deviations of the intensity or liking ratings were smaller for the younger age groups, consistent with previous findings. These results suggest that individual variations in texture perception and liking diminish as individuals become older, which might be related to a reduction in sensory function sensitivity due to aging.

Gender partially affected the texture perception and liking of the cooked rice or wheat bread samples. Compared to females, males exhibited higher intensity ratings of textural attributes in both cooked rice and wheat bread samples, except for hardness intensity for wheat bread samples. The overall liking of wheat bread samples was higher in males than in females, but no such gender difference in overall liking was observed in the cooked rice samples. Gender did not affect individual variations in the intensity ratings of texture attributes or influence JAR ratings for cooked rice or wheat bread samples.

The impact of consumption frequency was limited with regard to the textural attributes and overall liking of cooked rice and wheat bread samples, as evidenced by mean ratings, their standard deviations, and responses on the JAR scale. For cooked rice, there was no significant difference in stickiness and chewiness intensity, and for wheat bread, there was no significant difference in moistness and softness intensity based on consumption frequency. There were no variation differences in the intensity of textural attributes or overall liking of each sample due to differences in consumption frequency, and the percentages of “too much” responses of the JAR scale for textural attributes were also minimal.

Since this study clearly shows how age group, gender, and consumption frequency differed regarding the texture perception or liking of the cooked rice and wheat bread samples, professionals in agricultural and food systems can employ the findings to optimize the breeding and post-harvest processing of rice or wheat and in product development, increasing consumer acceptance and sales of the target products. Additionally, product developers and sensory professionals can gain a better understanding of how to develop cooked rice or wheat bread products for their target consumers, such as older adults, females or males, and frequent or infrequent eaters.

However, it would be interesting to conduct further research with cooked rice or wheat bread samples that have a wider variety of textural properties. Furthermore, given previous research indicating that cultural or regional background influence the texture perception or liking in cooked rice or bread, it would be interesting to understand how these variables modulate the intensity of textural attributes and overall liking of the test samples.

## Figures and Tables

**Table 1:** Participants’ demographics (*n* = 346).

Category	Subcategory	Frequency	Percentage (%)
Age group	20 to 29 years old	87	25.1
	30 to 49 years old	88	25.4
	50 to 69 years old	89	25.7
	70 to 79 years old	82	23.7
Gender	Females	172	49.7
	Males	174	50.3
Consumption frequency of cooked rice per week	<7 times (Low) ^1^	50	14.5
	7 times (Low)	79	22.8
	8 to 13 times (Medium)	38	11.0
	14 times (Medium)	114	32.9
	15 to 20 times (High)	25	7.2
	21 times (High)	40	11.6
Consumption frequency of wheat bread per week	<7 times (Low)	178	51.4
	7 times (Medium)	71	20.5
	8 to 13 times (Medium)	65	18.8
	14 times (High)	10	2.9
	15 to 20 times (High)	17	4.9
	21 times (High)	5	1.4

^1^ For the consumption frequency of cooked rice (or wheat bread) per week, its subcategories were classified into “Low”, “Medium”, and “High”.

**Table 2:** *F*-values (*p*-value) of the three-way analysis of variance (ANOVA), treating “age group”, “gender”, and “consumption frequency” as fixed effects, for textural attribute intensities and overall liking of cooked rice samples.

	Age Group (A)	Gender (G)	Consumption Frequency (C)	A × G	A × C	G × C
Hardness	6.44 (<0.001)	8.12 (0.004)	0.99 (0.37)	0.91 (0.44)	3.46 (0.002)	1.00 (0.37)
Stickiness	1.74 (0.16)	8.17 (0.004)	4.01 (0.02)	0.33 (0.80)	1.54 (0.16)	0.06 (0.94)
Chewiness	3.10 (0.03)	9.47 (0.002)	3.24 (0.04)	0.85 (0.47)	1.31 (0.25)	0.47 (0.63)
Overall liking	6.51 (<0.001)	2.87 (0.09)	1.89 (0.15)	0.43 (0.73)	1.52 (0.17)	0.48 (0.62)

**Table 3:** *F*-values (*p*-value) of the three-way analysis of variance (ANOVA), treating “age group”, “gender”, and “consumption frequency” as fixed effects, regarding standard deviations of the intensity or liking ratings of cooked rice samples.

	Age Group (A)	Gender (G)	Consumption Frequency (C)	A × G	A × C	G × C
Hardness	1.99 (0.12)	1.80 (0.18)	0.43 (0.65)	2.47 (0.06)	0.56 (0.76)	0.37 (0.69)
Stickiness	10.43 (<0.001)	0.27 (0.61)	1.56 (0.21)	1.35 (0.26)	0.95 (0.46)	0.07 (0.94)
Chewiness	8.69 (<0.001)	0.19 (0.66)	0.05 (0.95)	0.56 (0.64)	0.35 (0.91)	0.10 (0.91)
Overall liking	12.74 (<0.001)	6.08 (0.01)	0.03 (0.97)	0.59 (0.62)	0.59 (0.74)	0.76 (0.47)

**Table 4:** Summary of multivariate linear regressions for predicting overall liking of cooked rice samples.

	Model R^2^ (*p*-Value)	*β* Coefficient ^1^ (*p*-Value)			
		Constant	Hardness	Stickiness	Chewiness
Age group					
20–29 years	0.31 (<0.001)	2.276 (<0.001)	0.184 (0.003)	N.S.	0.497 (<0.001)
30–49 years	0.38 (<0.001)	1.049 (0.005)	0.226 (<0.001)	0.117 (0.048)	0.508 (<0.001)
50–69 years	0.55 (<0.001)	0.983 (0.001)	0.150 (0.003)	0.162 (0.013)	0.604 (<0.001)
70–79 years	0.38 (<0.001)	2.324 (<0.001)	N.S.	N.S.	0.662 (<0.001)
Gender					
Females	0.44 (<0.001)	1.185 (<0.001)	0.187 (<0.001)	0.126 (0.002)	0.562 (<0.001)
Males	0.38 (<0.001)	1.882 (<0.001)	0.179 (<0.001)	N.S.	0.551 (<0.001)
Consumption frequency					
Low	0.34 (<0.001)	1.659 (<0.001)	0.216 (<0.001)	0.101 (0.043)	0.448 (<0.001)
Medium	0.46 (<0.001)	1.433 (<0.001)	0.195 (<0.001)	N.S.	0.626 (<0.001)
High	0.43 (<0.001)	1.749 (<0.001)	N.S.	0.190 (0.007)	0.596 (<0.001)

^1^ Unstandarized *β*-coefficient; N.S.; No significance at *p* < 0.05.

**Table 5:** Percentages (mean drop) of “too little” or “too much” responses on the 5–point Just–About-Right scale for hardness, stickiness, and chewiness of cooked rice samples as a function of age group, gender, and consumption frequency.

	Hardness		Stickiness		Chewiness	
	Too Little ^1^	Too Much ^2^	Too Little	Too Much	Too Little	Too Much
Age group						
20–29 years	43.30% (0.98)	11.83% (0.44)	31.42% (0.39)	24.52% (0.77)	42.53% (0.95)	13.03% (0.81)
30–49 years	36.74% (0.82)	16.29% (0.87)	34.47% (1.48)	16.67% (0.59)	43.18% (1.10)	12.50% (-0.05)
50–69 years	33.33% (0.82)	15.36% (0.71)	33.33% (0.87)	17.98% (0.36)	36.70% (0.95)	14.98% (0.12)
70–79 years	24.80% (0.96)	20.33% (0.90)	28.86% (1.10)	16.26% (0.36)	30.08% (1.08)	19.92% (0.36)
Gender						
Females	34.11% (0.92)	17.25% (0.75)	34.11% (1.05)	16.86% (0.53)	39.92% (1.16)	14.15% (0.51)
Males	35.25% (0.93)	14.56% (0.74)	30.08% (0.91)	20.88% (0.59)	36.59% (0.93)	15.90% (0.08)
Consumption frequency						
Low	34.87% (1.16)	17.95% (0.64)	33.85% (0.93)	19.49% (0.20)	40.00% (1.08)	18.46% (0.15)
Medium	33.59% (0.66)	16.02% (0.98)	28.68% (0.80)	18.35% (0.54)	37.73% (0.95)	13.70% (0.39)
High	35.53% (1.04)	14.91% (0.65)	34.21% (1.15)	19.08% (0.73)	37.94% (1.14)	14.69% (0.29)

^1^ The “much too little” and “too little” responses were combined into “too little”. ^2^ The “much too much” and “too much” responses were combined into “too much”.

**Table 6:** *F*-values (*p*-value) of the three-way analysis of variance (ANOVA), treating “age group”, “gender”, and “consumption frequency” as fixed effects, for textural attribute intensities and overall liking of wheat bread samples.

	Age Group (A)	Gender (G)	Consumption Frequency (C)	A × G	A × C	G × C
Hardness	3.32 (0.02)	4.03 (0.045)	1.43 (0.24)	4.30 (0.005)	1.23 (0.29)	0.27 (0.76)
Moistness	16.14 (<0.001)	19.88 (<0.001)	6.46 (0.002)	3.40 (0.02)	2.02 (0.06)	1.26 (0.28)
Chewiness	3.06 (0.03)	18.55 (<0.001)	1.86 (0.16)	2.35 (0.07)	0.75 (0.61)	2.35 (0.10)
Softness	7.16 (<0.001)	23.10 (<0.001)	3.39 (0.03)	2.61 (0.05)	0.95 (0.46)	2.53 (0.08)
Overall liking	7.05 (<0.001)	24.24 (<0.001)	6.96 (<0.001)	1.01 (0.39)	0.52 (0.80)	2.50 (0.08)

**Table 7:** *F*-values (*p*-value) of the three-way analysis of variance (ANOVA), treating “age group”, “gender”, and “consumption frequency” as fixed effects, regarding standard deviations of the intensity or liking ratings of wheat bread samples.

	Age Group (A)	Gender (G)	Consumption Frequency (C)	A × G	A × C	G × C
Hardness	9.74 (<0.001)	0.22 (0.64)	0.14 (0.87)	0.37 (0.78)	2.09 (0.05)	2.15 (0.12)
Moistness	20.04 (<0.001)	1.82 (0.18)	1.53 (0.22)	1.19 (0.31)	3.67 (0.002)	0.17 (0.84)
Chewiness	10.37 (<0.001)	1.19 (0.28)	0.10 (0.90)	0.47 (0.70)	2.56 (0.02)	0.73 (0.48)
Softness	16.40 (<0.001)	0.71 (0.40)	1.28 (0.28)	2.10 (0.10)	3.24 (0.004)	0.18 (0.84)
Overall liking	20.49 (<0.001)	1.56 (0.21)	0.10 (0.91)	0.64 (0.59)	3.71 (0.001)	0.07 (0.93)

**Table 8:** Summary of multivariate linear regressions for predicting overall liking of wheat bread samples.

	Model R^2^ (*p*-Value)	*β* Coefficient ^1^ (*p*-Value)				
		Constant	Hardness	Moistness	Chewiness	Softness
Age group						
20–29 years	0.66 (<0.001)	1.781 (<0.001)	−0.187 (<0.001)	0.418 (<0.001)	0.186 (<0.001)	0.355 (<0.001)
30–49 years	0.73 (<0.001)	1.274 (0.006)	−0.157 (0.003)	0.406 (<0.001)	0.202 (<0.001)	0.387 (<0.001)
50–69 years	0.62 (<0.001)	1.919 (<0.001)	−0.245 (<0.001)	0.372 (<0.001)	0.281 (<0.001)	0.314 (<0.001)
70–79 years	0.35 (<0.001)	3.106 (<0.001)	−0.290 (<0.001)	0.223 (<0.001)	0.184 (0.002)	0.358 (<0.001)
Gender						
Females	0.72 (<0.001)	2.036 (<0.001)	−0.233 (<0.001)	0.368 (<0.001)	0.160 (<0.001)	0.411 (<0.001)
Males	0.53 (<0.001)	2.276 (<0.001)	−0.258 (<0.001)	0.327 (<0.001)	0.283 (<0.001)	0.291 (<0.001)
Consumption frequency						
Low	0.61 (<0.001)	2.340 (<0.001)	−0.277 (<0.001)	0.345 (<0.001)	0.216 (<0.001)	0.346 (<0.001)
Medium	0.65 (<0.001)	1.845 (<0.001)	−0.197 (<0.001)	0.343 (<0.001)	0.199 (<0.001)	0.394 (<0.001)
High	0.66 (<0.001)	2.365 (<0.001)	−0.277 (<0.001)	0.429 (<0.001)	0.331 (<0.001)	N.S.

^1^ Unstandarized *β*-coefficient. N.S. = No significance at *p* < 0.05.

**Table 9:** Percentages (mean drop) of “too little” or “too much” responses on the 5–point Just-About-Right scale for hardness, moistness, chewiness, and softness of wheat bread samples as a function of age group, gender, and consumption frequency.

	Hardness		Moistness		Chewiness		Softness	
	Too Little ^1^	Too Much ^2^	Too Little	Too Much	Too Little	Too Much	Too Little	Too Much
Age group								
20–29 years	6.03% (1.19)	52.30% (3.13)	64.08% (3.07)	4.02% (0.22)	37.64% (2.03)	17.82% (1.57)	56.90% (2.89)	5.17% (0.13)
30–49 years	8.05% (0.89)	56.90% (2.94)	67.82% (3.30)	2.30% (−0.39)	49.71% (2.77)	12.36% (2.07)	66.95% (3.55)	5.17% (1.37)
50–69 years	12.22% (1.21)	50.00% (2.56)	56.39% (2.64)	7.78% (0.17)	42.50% (2.46)	17.50% (1.38)	54.17% (2.99)	11.39% (1.03)
70–79 years	16.16% (0.98)	33.84% (1.36)	46.34% (1.55)	9.45% (0.45)	35.37% (1.64)	19.21% (1.40)	36.89% (1.84)	16.16% (0.73)
Gender								
Females	8.43% (1.26)	52.76% (2.75)	62.79% (2.98)	5.09% (0.58)	47.53% (2.45)	14.97% (2.07)	62.06% (3.14)	7.12% (0.76)
Males	12.64% (1.01)	44.25% (2.37)	54.89% (2.34)	6.61% (0.08)	35.35% (2.05)	18.39% (1.15)	45.98% (2.54)	11.64% (0.91)
Consumption frequency								
Low	10.11% (1.25)	49.16% (2.55)	61.66% (2.58)	4.92% (0.39)	41.57% (2.25)	16.71% (2.00)	55.06% (2.77)	8.43% (1.23)
Medium	11.03% (0.96)	47.06% (2.63)	56.62% (2.79)	5.52% (0.59)	42.10% (2.40)	15.26% (1.24)	53.68% (2.96)	9.56% (0.78)
High	10.94% (0.95)	50.78% (2.70)	52.34% (2.69)	12.50% (−0.42)	37.50% (1.91)	22.66% (0.67)	49.22% (2.84)	14.06% (−0.08)

^1^ The “much too little” and “too little” responses were combined into “too little”. ^2^ The “much too much” and “too much” responses were combined into “too much”.

## Data Availability

The data are not publicly available due to the Institutional Review Board protocol guideline.
